# Syndromes and anomalies associated with cleft

**DOI:** 10.4103/0970-0358.57187

**Published:** 2009-10

**Authors:** R. Venkatesh

**Affiliations:** Department of Plastic Surgery, Sri Ramachandra University, Chennai, India

**Keywords:** Adult patients, Cleft lip, Cleft palate, Premaxilla

## Abstract

Orofacial clefts are one of the commonest birth defects, and may be associated with other congenital anomalies. The majority of these orofacial clefts are nonsyndromic. A significant percentage of these clefts both syndromic and non-syndromic may have associated anomalies. Apart from reviewing other studies, this article also analyses a study of associated anomalies from a tertiary cleft centre in India.

## INTRODUCTION

Every congenital structural defect in the body represents an inborn error in morphogenesis and may affect one or more systems. Various authors have reported incidence of associated anomalies varying from as low as 4.3%[1] to as high as 63.4%.[2]

Organogenesis takes place on Days 25-28 of intrauterine life. Any insult like environmental, developmental or nutritional to the embryo during this period will lead to malformations. Associated anomalies are classified according to the organs affected.

In general, most congenital anomalies can be divided into three types **a*) Disruptions:*** A rare anomaly related to breakdown of the original normal foetal developmental process, e.g. craniofacial cleft resulting from amniotic bands. ***b) Deformations:*** These occur secondary to mechanical forces leading to anomalies of a lesser degree when compared to disruption, e.g. club foot, cleft palate, Pierre Robin sequence etc. ***c) Malformations*:** A morphologic defect in an organ from an intrinsically abnormal developmental process, e.g. polydactyly, congenital heart anomalies, cleft lip etc.

However, with the present advancement in embryology and genetics, and its correlations, the associated anomalies need to be differentiated from syndromes, from sequences and associations in patients with multiple congenital anomalies. They are generally described in four categories.

## MONOGENIC SYNDROME

When the anomalies are aetiologically related and due to a single gene, the constellation of associated anomalies constitutes a *monogenic syndrome.* A review by Gorlin described 72 monogenic syndromes involving Oral clefts (OCs). A follow-up report by Cohen[3] identified 154 monogenic syndromes and, more recently, 487 were identified in the 2001 version of the London Dysmorphology database (Winter and Baraitser, 2001).[4] Monogenic syndromes include Van der Woude with most of these cases linked to Chromosome 1q32-q41 and Treacher Collins (an autosomal dominant) syndrome.

## CHROMOSOMAL SYNDROME

These syndromes involve a clinically significant structural and/or numerical chromosomal abnormality. The deletion of Chromosome 22q11.2, for example, causes the Velocardiofacial syndrome (Shprintzen syndrome-Cleft palate, cardiac anomalies, typical facies, and learning disabilities). Trisomies 13 and 18, and the 4p- are other chromosomal abnormalities leading to different syndromes often found with oral clefts.[5]

### Sequence

When the associated anomalies are due to a single known or presumed structural defect, they are termed *sequence*. The most common sequence observed with oral clefts is the Pierre Robin sequence, which is characterized by mandibular deficiency, cleft palate, and upper airway obstruction. It was named Pierre Robin syndrome, anomalad or complex, but since it is regarded as a series of events during embryology like micrognathic jaw leading to cleft palate, it is now known as a sequence.[6]

### Association

The non-random occurrence of several morphologic defects not identified as a syndrome or a sequence is an *association*. Oral clefts are frequently associated with congenital heart defects. The cause of these associations is unknown. This category is also called “multiple congenital anomaly”.[7]

However, as the genes that cause oral clefts are identified, a number of sequences and associations will be reclassified as monogenic syndromes.

#### Clinical evaluation

The approach in dealing with any given anomaly including clefts is to study the associated defects. Due to variation in the timing of the development of abnormalities, follow-up until the child is four or five years of age is essential. The general guidelines for the evaluation of individuals with Cleft lip and palate and Cleft palate (CL/P and CP) in order to identify syndromes are:

- Thorough clinical examination, preferably by geneticist or dysmorphologist.
- Comprehensive medical history: Description of the cleft, antenatal history, birth history, developmental history, and family history.
- Physical examination: measurement of weight, length or height, and occipitofrontal circumference, identification of anomalies of eyes, ears, heart, extremities and also to look for associated preauricular tags, lip pits, epicanthal folds.
- Documentation by photographs of all affected individuals and first-degree relatives.
- Necessary laboratory and radiological evaluations.

## LITERATURE REVIEW

Prevalence of associated anomalies with orofacial clefts can differ among different populations. At birth, black population has lower prevalence of oral clefts compared to whites; Sullivan[8] found that oral clefts in the black population are more commonly associated with clubfoot and polydactyly compared to other ethnic population. The explanation given is that polydactyly and clubfoot are more prevalent in blacks than in whites.[9]

Population-based studies will be more appropriate. In addition, one should also consider spontaneous abortions, elective terminations, stillborn foetuses, and babies that died shortly after birth to get the true numbers of associated anomalies. It is also essential that every child should be thoroughly examined immediately after birth for the associated anomalies, because children with severe malformation may not survive long. Jensen[10] recognized that, and because they did not study stillbirths and early deaths, their figure of 4.5% probably considerably underestimates the true frequency. We have mentioned here some important studies from different countries.

### 

#### Sweden

Population-based study showed that 1% of patients with oral cleft had associated malformations that either required follow-up or treatment. Associated malformations were more frequent in infants who had both cleft lip and palate (28%) than in infants with isolated cleft palate (22%) or infants with isolated cleft lip (8%). Malformations of the limbs or vertebral column were the most common anomalies and accounted for 33% of all associated defects. 24% of associated malformations were of the cardiovascular system and congenital heart disease was the most common isolated associated malformation. 15% of all associated malformations were multiple and they were frequently associated with mental retardation or chromosomal anomalies. 22% of infants with associated malformations were born preterm, compared with an expected 5% incidence of preterm delivery in Sweden.[11]

#### France

This study was based on 238,942 consecutive births of known outcome registered by the registry of congenital anomalies, 36.7% are reported as having associated malformations. These were most frequent in infants with cleft palate only (46.7%), as against infants with cleft lip and palate (36.8%), or infants with cleft lip only (13.6%). Malformations in the central nervous system and in the skeletal system were the anomalies most commonly associated. Next in frequency were malformations in the urogenital and cardiovascular systems. Although prenatal ultrasonic tests were carried out, a success rate of only 31.6% is reported in diagnosis.[12]

#### China

In multiple institutional-based studies from China, 7812 patients having cleft lip and or palate were studied for associated malformations. Overall, 2.89% of orofacial patients had associated anomalies. The frequency of associated malformations in CLP (3.35%) was higher than CL (2.24%) and CP (2.22%)[13]

#### Iran

1669 consecutive cases of cleft lip and or palate were analyzed in a referral centre. Overall 7.73% of the cases had associated anomalies, of which 4.5% of CLP children had associated anomalies compared to 3.1%in cases with CP. The majority of the associated anomalies were skeletal (32%) and in the head and neck region (18%).[14]

#### Tertiary Care centre in India

2600 clefts patients were analyzed in a study from the cleft and craniofacial centre of Sri Ramachandra University. Of the 2600 patients, 198 had associated anomalies. Associated anomalies were more frequent in patients with cleft lip and palate (32%) than in patients with cleft lip alone (11%) or patients with cleft palate alone (22%). The organs most commonly involved with associated anomalies in the order of decreasing incidence are Eye (49), Ear (34), Heart (33), Upper Limb (22), Lower Limb (17), Genitals (22), Mandible (17), Mental retardation (12), Craniofacial clefts (10), Skull (10), Tongue (9), Growth Retardation (2), Skin and Hair (1).

A significant percentage of patients (36%, 72 / 198) with associated anomalies were syndromic. The common syndromes were Van der Woude syndrome, Median facial dysplasia syndrome and Pierre Robin Sequence.[15]

## COMMON SYNDROMES ASSOCIATED WITH ORO-FACIAL CLEFT (OCF)

### 

#### Van der Woude Syndrome [Figure 1]

**Figure 1:**
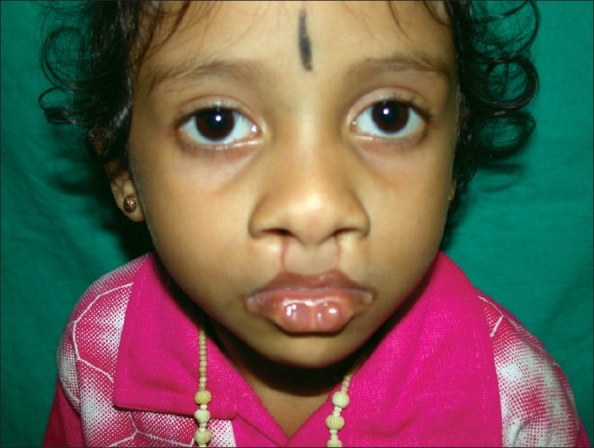
Van der Woude syndrome with lower lip pits

It is one of the commonest syndromes associated with oral cleft. It is transmitted as an autosomal dominant and lower lip pits are the hallmark. These pits are located bilaterally in the lower lip at the junction of dry and wet vermilion and they are either oval or transverse in shape. Pits traverse the underlying orbicularis muscle and end in a blind pouch on the buccal side and communicate with minor salivary glands. The associated features are hypodontia, missing maxillary or mandibular second premolar teeth, absent maxillary lateral incisor and ankyloglosia. Other extra-oral manifestations though rare include accessory nipples, congenital heart defects, Hirschsprung disease and popliteal web.[16]

#### Pierre Robin sequence [Figure 2]

**Figure 2:**
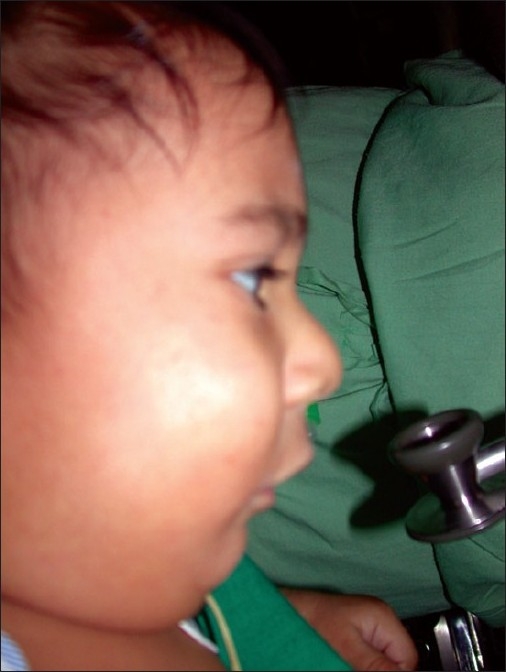
Pierre Robin sequence

In 1926, Pierre Robin published a case with a complete syndrome with triad of glossoptosis, micrognathia and airway obstruction. Although cleft is not included in the triad, it is frequently associated and may aggravate the obstruction due to tongue fall. Although many theories have been proposed for the sequence, the mechanical theory is the most accepted. The initial event is mandibular hypoplasia between the seventh and eleventh weeks of gestation, which keeps the tongue high in the oral cavity preventing closure of palatal shelves resulting in formation of classic inverted U-shaped cleft palate. Oligohydramnios also plays a role because lack of amniotic fluid leads to deformation of the chin and subsequent impaction of the tongue between palatal shelves. The frequency of occurrence of various deformities are Micrognathia (91.7%), Glossoptosis (70-85%) or Macroglossia and Ankyloglossia (10–15 %) and Cleft Palate (14 %). Occasionally a bifid or double uvula with an occult submucous cleft can be present. Airway obstruction due to tongue fall results in failure to thrive and is a serious problem in these patients. A great degree of suspicion is required to diagnose this condition, and management includes proper feeding advice, positioning the baby and early surgical intervention.[17,18]

#### Velocardiofacial syndrome [Figure 3]

**Figure 3:**
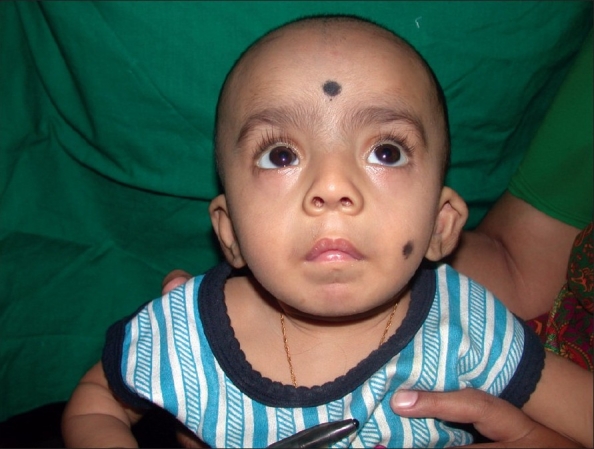
Velocardiofacial syndrome with typical facies

Velocardiofacial syndrome (VCFS) is an autosomal dominant condition and is associated with Chromosome 22q abnormality, as a result of a sub-microscopic deletion on the long arm of Chromosome 22 in the“q11”region (deletion22q11). It was described by Dr. Robert J. Shprintzen. It occurs in approximately one in 2000 live births[19] and is the most common sub-microscopic deletion syndrome. There are more than 100 physical phenotypic features reported, as VCFS affects every major system in the body. The most common features are cleft palate, cardiac anomaly, characteristic facial appearance (vertical maxillary excess, malar flattening, relative mandibular retrusion, narrow palpebral fissure and small ears), minor learning problems, speech and feeding problems. There is a close association between VCFS and DiGeorge syndrome which includes small or absent thymus, tonsils, adenoids and hypocalcaemia. These children may have medial displacement of the carotid artery over the cervical vertebrae and this should be borne in mind while planning any pharyngeal surgery like pharyngeal flap for Velo pharyngeal incompetence (VPI) correction. The majority of these patients will need support for their learning problems.[19]

#### Median facial dysplasia [Figure 4]

**Figure 4:**
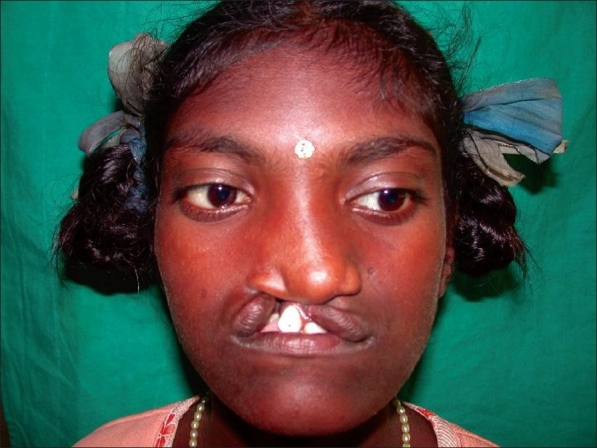
Median facial dysplasia

Median facial dysplasia is a unique, distinct, definable group of patients characterized by midline facial deficiencies in the presence of a unilateral or bilateral cleft lip with or without cleft palate.[20] The midline hypoplasia may extend into the midline structure of the brain like corpus callosum. If head circumference is <90% of normal, these patients may have associated anomalies of the brain, especially frontal corpus callosum. It is obvious that they will have compromised development of midface resulting in very early dish face, Class III occlusion and severe maxillary hypoplasia. Early recognition of these subgroups of patients helps to plan the course of treatment.

## CONCLUSION

Recognition of the associated syndromes and anomalies with the oral cleft is essential to assess the problem and risk faced by the child and for counselling the parents. Proper knowledge and details of anomalies associated with OFC will help to provide necessary treatment and improve survival of these children. Proper epidemiology, dysmorphology assessment and genetic study may lead researchers to the identification of the causative agent.
